# High-throughput DNA extraction strategy for fecal microbiome studies

**DOI:** 10.1128/spectrum.02932-23

**Published:** 2024-05-15

**Authors:** Heidi Isokääntä, Natalie Tomnikov, Sanja Vanhatalo, Eveliina Munukka, Pentti Huovinen, Antti J. Hakanen, Teemu Kallonen

**Affiliations:** 1 Infections and Immunity Unit, Institute of Biomedicine, University of Turku, Turku, Finland; 2 Centre for Population Health Research, University of Turku, Turku, Finland; 3 Department of Clinical Microbiology, Tyks Laboratories, Turku University Hospital, Turku, Finland; 4 Clinical Microbiome Bank, Microbe Center, Turku University Hospital and University of Turku, Turku, Finland; 5 Division of Digestive Surgery and Urology, Turku University Hospital, Turku, Finland; University of Michigan-Ann Arbor, Ann Arbor, Michigan, USA

**Keywords:** DNA extraction, high throughput, gut microbiome, fecal sample, method development, sample preservative

## Abstract

Microbiome studies are becoming larger in size to detect the potentially small effect that environmental factors have on our gut microbiomes, or that the microbiome has on our health. Therefore, fast and reproducible DNA isolation methods are needed to handle thousands of fecal samples. We used the Chemagic 360 chemistry and Magnetic Separation Module I (MSMI) instrument to compare two sample preservatives and four different pre-treatment protocols to find an optimal method for DNA isolation from thousands of fecal samples. The pre-treatments included bead beating, sample handling in tube and plate format, and proteinase K incubation. The optimal method offers a sufficient yield of high-quality DNA without contamination. Three human fecal samples (adult, senior, and infant) with technical replicates were extracted. The extraction included negative controls (OMNIgeneGUT, DNA/RNA shield fluid, and Chemagic Lysis Buffer 1) to detect cross-contamination and ZymoBIOMICS Gut Microbiome Standard as a positive control to mimic the human gut microbiome and assess sensitivity of the extraction method. All samples were extracted using Chemagic DNA Stool 200 H96 kit (PerkinElmer, Finland). The samples were collected in two preservatives, OMNIgeneGUT and DNA/RNA shield fluid. DNA quantity was measured using Qubit-fluorometer, DNA purity and quality using gel electrophoresis, and taxonomic signatures with 16S rRNA gene-based sequencing with V3V4 and V4 regions. Bead beating increased bacterial diversity. The largest increase was detected in gram-positive genera *Blautia,* Bifidobacterium, and *Ruminococcus*. Preservatives showed minor differences in bacterial abundances. The profiles between the V3V4 and V4 regions differed considerably with lower diversity samples. Negative controls showed signs from genera abundant in fecal samples. Technical replicates of the Gut Standard and stool samples showed low variation. The selected isolation protocol included recommended steps from manufacturer as well as bead beating. Bead beating was found to be necessary to detect hard-to-lyse bacteria. The protocol was reproducible in terms of DNA yield among different stool replicates and the ZymoBIOMICS Gut Microbiome Standard. The MSM1 instrument and pre-treatment in a 96-format offered the possibility of automation and handling of large sample collections. Both preservatives were feasible in terms of sample handling and had low variation in taxonomic signatures. The 16S rRNA target region had a high impact on the composition of the bacterial profile.

**IMPORTANCE:**

Next-generation sequencing (NGS) is a widely used method for determining the composition of the gut microbiota. Due to the differences in the gut microbiota composition between individuals, microbiome studies have expanded into large population studies to maximize detection of small effects on microbe–host interactions. Thus, the demand for a rapid and reliable microbial profiling is continuously increasing, making the optimization of high-throughput 96-format DNA extraction integral for NGS-based downstream applications. However, experimental protocols are prone to bias and errors from sample collection and storage, to DNA extraction, primer selection and sequencing, and bioinformatics analyses. Methodological bias can contribute to differences in microbiome profiles, causing variability across studies and laboratories using different protocols. To improve consistency and confidence of the measurements, the standardization of microbiome analysis methods has been recognized in many fields.

## INTRODUCTION

Due to better understanding of the microbial communities, new links are constantly discovered between the human microbiome and health (1–7). Host and gut microbiota form a complex and active ecosystem, which harbors an enormous variety of microbes forming a community, playing important roles, e.g., in our metabolism and immune system (8–12). During the early stages of life, the gut microbiome is typically low in diversity. In adulthood, the gut microbiome is relatively stable, yet still susceptible to changes caused by life events. It can be stated that a stable and diverse microbiome is a vital factor of an individual’s health and well-being. At an older age, the diversity of microbiome decreases and becomes unstable (13–15). Furthermore, due to the high variation in gut microbiome between individuals, defining a global, unequivocal healthy gut microbiome is challenging (6).

Next-generation sequencing (NGS) is a widely used method for determining the genetic composition of the gut microbiota. Due to the great differences in the composition of the gut microbiota between individuals, microbiome studies have expanded into large populations to maximize detection of these small effects of microbe–host interactions. Thus, the demand for a rapid, efficient, and reliable microbial profiling is continuously increasing, making the optimization of high-throughput 96-format DNA extraction integral prior to NGS-based downstream applications (16–19).

Metagenomics has an indispensable role at many stages of microbiome-based product development, identification of microbial targets and clinical trials. However, workflows and experimental protocols are complex and prone to bias and errors at all steps, from sample collection and storage (20, 21) to DNA extraction (22–24), primer selection (25–27) and sequencing, and bioinformatics analyses (28–31). Previous studies have shown that multiple preservatives can be feasible in sample collection and compatible for DNA isolation (32, 33). Some of the challenges may occur due to differences in bacterial species characteristics, such as persistent cell walls or envelopes. Gram-positive bacteria are typically considered hard-to-lyse because of the thicker peptidoglycan cell wall. Mechanical lysis has been found to increase DNA yield but predispose to DNA shearing. With an ideal DNA extraction method, all bacteria should be recovered equally well (34–36).

Methodological bias can contribute to remarkable differences in observed microbiome profiles, causing variability in results across studies and laboratories with different protocols (37–39). To improve consistency and confidence in the accuracy of the measurements, the standardization of microbiome analysis methods has been recognized as an urgent need by academic, diagnostic, industrial, and regulatory sectors (40–43).

### Study aims

This study aimed to investigate what is the best practice for automated DNA extraction for downstream sequencing applications and handling of large sample collections in microbiome research and clinical microbiology. The best practice needs to offer sufficient yield of high-quality DNA, minimized contamination and human error and low variability in the results. This study focuses on the impact of human fecal sample collection (preservative), pre-treatment of DNA extraction, and further, choice of 16S rRNA gene hypervariable region to microbiome results.

## MATERIALS AND METHODS

**Fig 1:**
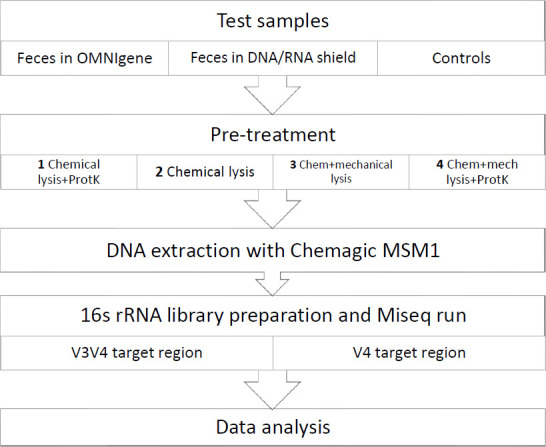
An overview of the study design. Controls included negative controls of extraction (lysis buffer) and preservatives (OMNIgeneGUT and DNA/RNA shield fluid), and Gut Standard as positive extraction controls.

The optimization workflow is represented in Fig. 1. Two commercial sample preservatives, namely OMNIgeneGUT (DNA Genotek, Canada) and DNA/RNA shield fluid (Zymo Research, USA), were tested in fecal sample collection. In addition, four different pre-treatment protocols were tested with all samples including bead beating (mechanical lysis) and proteinase K incubation. Comparison of two 16S rRNA target regions (V3V4 and V4) was also carried out.

### Test samples

Human fecal samples from healthy infant, adult, and senior volunteers (*n* = 3) were simultaneously collected in both OMNIgeneGUT and DNA/RNA shield fluid tubes. After 3 d of storage at room temperature, simulating transport from home to the laboratory by mail, the samples were frozen at −80°C. DNA extraction was performed 2 wk after freezing. The ZymoBIOMICS Gut Microbiome Standard (Zymo Research, USA) was used as a positive extraction control to mimic a human gut microbiome with a known microbial composition. The commercial Gut Standard was used to investigate whether the results were close to the theoretical composition, and all taxa were equally extracted. Manufacturer uses the V3V4 region of the 16S gene to assign reference abundances for the standard. A kit lysis buffer (Chemagic Lysis Buffer 1) and OMNIgene fluid and RNA/DNA shield fluid were used as negative controls. Altogether, 192 samples were sequenced (40 repeats of adult sample, 32 repeats of infant sample, 30 repeats of senior sample, 24 repeats of Gut Standard, 60 negative controls, and 6 PCR controls).

### Pre-treatment and DNA extraction

Microbial DNA was extracted from adult and infant fecal samples as well as the ZymoBIOMICS Gut Microbiome Standard and negative controls using a DNA Stool 200 H96 kit (PerkinElmer, Finland) with Magnetic Separation Module I (MSM I) extraction robot (PerkinElmer, Finland). Different sample preparation procedures, including bead beating and chemical lysis, were applied to the samples to assess the impact of the sample lysis and homogenization. Three technical replicates of the adult and senior samples and two technical replicates of the infant sample were extracted. The same extraction also included negative controls and the ZymoBIOMICS Gut Standard. Negative controls were placed between fecal samples to detect cross-contamination. The Gut Standard was used to assess the sensitivity of the extraction method.

Pre-treatment procedures were modified from the manufacturer’s protocol “Purification Protocol for Human Feces Material Using the Chemagic Magnetic Separation Module I.” The following volumes of reagents and samples were used in every pre-treatment group. Lysis Buffer 1 (800 µL) was added to 200 µL of the fecal sample. Subsequently, 925 µL of Lysis Buffer 1 was added to 75 µL of the ZymoBIOMICS Gut Standard. For negative controls (OMNIgene and DNA/RNA shield fluid), the volume of 800 µL of Lysis Buffer 1 was added to 200 µL of each collection fluid. Finally, for Lysis Buffer 1 extraction control, 1 mL of buffer was used. The pre-treatment was divided into four groups (Table 1).

**TABLE 1:** Pre-treatment groups of DNA extraction

Group	Lysis	Pre-treatment
1	Chemical	Manufacturer’s protocol; incubation with proteinase K
2	Chemical	Manufacturer’s protocol; incubation without proteinase K
3	Chemical + mechanical	Bead plate + tissue lyser 15 Hz; 2 × 5 min
4	Chemical + mechanical	Bead plate + tissue lyser 15 Hz; 2 × 5 min + proteinase K incubation

Group 1 included MSM I manufacturer’s original protocol with proteinase K incubations. After the addition of the lysis buffer, the tubes were vortexed and 15 µL of proteinase K was added, incubated in a thermo shaker at 70°C for 10 min, followed by incubation at 95°C for 5 min. Samples were centrifuged at a high speed for 5 min. The lysate (800 µL) was then transferred to a sample plate, and the extraction proceeded according to the manufacturer’s protocol.

Group 2 included MSM I manufacturer’s original protocol without proteinase K incubations.

Group 3 included bead beating with a PowerBead Pro Plates (Glass beads 0.1 mm) and a TissueLyser II (Qiagen, USA). The fecal samples, the Gut Standard, negative controls, and lysis buffer were added to the bead plates, and the bead plate was sealed with a sealing film. The plate was shaken in the TissueLyser II at 15 Hz for 5 min twice. Next, the plate was centrifuged at 4,500 × *g* for 6 min, and 800 µL of the lysate was transferred to the sample plate; the extraction proceeded according to the manufacturer’s protocol.

Group 4: bead beating with a bead plate and the TissueLyser II was combined with proteinase K incubations. The plate was shaken in the same way as mentioned in group 3. After shaking, the plate was centrifuged, and 800 µL of the lysate was transferred to 2-mL screw cap tubes with 15 µL of proteinase K. The tubes were vortexed and incubated as mentioned in group 1. The tubes were briefly placed in a spinner, and the lysate was transferred to the sample plate.

After the extraction step, the DNA concentration was measured with a Qubit 2.0 Fluorometer (Thermo Fisher Scientific, USA) using a Qubit dsDNA High Sensitivity Assay kit (Thermo Fisher Scientific, USA). DNA integrity was evaluated by 1% TBE agarose gel electrophoresis. The DNA was divided into two 100-µL aliquots and stored at −80°C. DNA extraction and a downstream analysis were performed with DNase-/RNase-free plastics.

### Library preparation and 16S sequencing

Microbial composition was determined by sequencing both V3V4 and V4 regions of the 16S ribosomal gene using a MiSeq platform (Illumina, USA). The sequence library was constructed according to the Illumina library preparation protocol (44) and V4 library with an in-house protocol (45).

For V4, two replicates of the fecal samples were sequenced. In the V4 library preparation, amplicon PCR and index PCR were combined (45). The DNA was diluted in PCR-grade water to 10 ng/µL prior to PCR. PCR was performed with KAPA HiFi High Fidelity PCR kit with dNTPs (Roche, USA). The desired concentration of each component was the following: 1x for 5x KAPA HiFi Fidelity Buffer, 0.3 mM for dNTP mix, 0.5 U for KAPA HiFi DNA polymerase and PCR-grade water. Sequences of the forward and reverse primers (0.3 µM) were 5′- AATGATACGGCGACCACCGAGATCTACAC -i5- TATGGTAATT-GTGTGCCAGCMGCCGCGGTAA-3′ (forward) and 5′- CAAGCAGAAGACGGCATACGAGAT -i7- AGTCAGTCAG-GCGGACTACHVGGGTWTCTAAT-3′ (reverse), where i5 and i7 indicate the sample specific indices. The concentration of template DNA was 50 ng. The final volume of the reaction was 25 µL. Combined PCR had following conditions: initial denaturation at 98°C for 4 min, followed by 30 cycles consisting of denaturation at 98°C for 20 s, annealing at 65°C for 20 s and extension at 72°C for 35 s, and with a final extension at 72°C for 10 min.

V3V4 sequencing included all replicates of the fecal samples. Sequencing also included positive and negative controls. The V3V4 protocol differed from Illumina’s recommendation for the final volumes of the PCR reaction and the DNA visualization procedures. Prior to PCR, the DNA samples were diluted to 2.5 ng/µL in PCR-grade water. Briefly, amplicon PCR included 2x KAPA HiFi HotStart ReadyMix (Roche, USA), Illumina amplicon forward and reverse primers (6.6 µM), PCR-grade water, and microbial DNA (16.5 ng). The final volume of the amplicon PCR reaction was 33 µL. Index PCR was performed according to Illumina’s instructions.

After PCR, 8 µL of the product was analyzed with 1.5% TAE agarose gel (120 V, 1 h). The concentration of the library samples was measured with a Qubit Fluorometer using a Qubit dsDNA High Sensitivity Assay kit. The 4 nM library pool was denatured, diluted to a concentration of 4 pM, and an 8% denaturized PhiX control (Illumina, USA) was added. The library samples were sequenced with a MiSeq Reagent kit v3, 600 cycles (Illumina, USA) on a MiSeq system with 2 × 300 base pair (bp) paired ends following the manufacturer’s instructions. The library samples were sequenced with an Illumina MiSeq Reagent kit v3 (600 cycles) on a MiSeq system with 2 × 250 bp paired ends following the manufacturer’s instructions. A positive plasmid control (DNA 7-mock) and a negative control (PCR-grade water) were included in library preparation to control the PCR.

### Bioinformatic methods and data visualization

The raw sequence data for both libraries were processed and analyzed with a CLC Microbial Genomics Module (CLC Genomics Workbench 21.0.3, Qiagen, USA). The workflows “Data quality control (QC) and operational taxonomic unit (OTU) clustering” including read trim and “Alpha and beta diversities” were used to analyze the data using default settings. The 16S read pairs were merged. The cutoff for the number of reads of the fecal samples was 200,000 sequences; however, negative controls were not filtered based on the number of reads. One infant sample from V3V4 sequencing was excluded due to the low number of reads (reads after trim, 193,762). An index and adapter trims from the 5′ end were performed for both libraries. Sequences were mapped using a SILVA 16S version 132 with a 97% similarity for OTU clustering. In the diversity analysis, low abundance OTUs were filtered (<100 reads), and OTUs were aligned using MUSCLE (MUltiple Sequence Comparison by Log-Expectation). A neighbor-joining tree was used to calculate alpha diversity. Chao1 and the total OTU number (observed OTUs) were selected to represent alpha diversity. Alpha diversity of the total OTU number represents richness; the number of species observed in each sample and Chao1 estimates the total richness that accounts for unobserved species (46). Two-sample *t*-test was used to test statistically significant difference in alpha diversity between pre-treatment groups. A rarefaction level of 78,948 was used for the alpha diversity. Beta diversity calculations were performed using the principal coordinate analysis (PCoA) with Jaccard and Bray-Curtis. OTU tables and alpha and beta diversities were exported to a RStudio 4.1.1 and GraphPad Prism 9.0.1 for data visualization. Differential abundances (DA) were analyzed with CLC Microbial Genomics Module and Deseq2 (47) in R environment. The reported DA results are consensus between the two methods mentioned above.

## RESULTS

### DNA yields

All pre-treatment methods yielded sufficient amount of DNA (>7 ng/µL) in the fecal samples. The non-bead-beating group 2 with both stabilizers produced the highest concentration for the adult samples. For senior samples, collected in OMNIgeneGUT, the highest concentration was observed in group 4 (bead beating and prot K). In infant samples, the highest concentration was achieved without bead beating (groups 1 and 2). DNA concentrations are shown in Table S1.

Adult fecal samples seemed to have the lowest deviation in concentrations across pre-treatment groups, whereas infant samples had the highest standard deviation. OMNIgeneGUT offered a little higher concentration with senior and infant samples than DNA/RNA shield, but with the adult sample, there was no marked difference. The expected concentration of the Gut Standard was 5 ng/µL according to the manufacturer. Different pre-treatments yielded, on average, 5.35 ng/µL of the standard (4.0–6.4 ng/µL) (Table S1).

The integrity of the DNA isolates was detected with gel electrophoresis, which showed that all fecal samples had a visible amount of DNA (Fig. S1). Senior samples in OMNI (“SO”) had some fragmentation. Correspondingly, those senior samples also had the highest DNA concentrations of all samples. Extraction controls (“EC”) were all pure.

### Gut Standard

**Fig 2:**
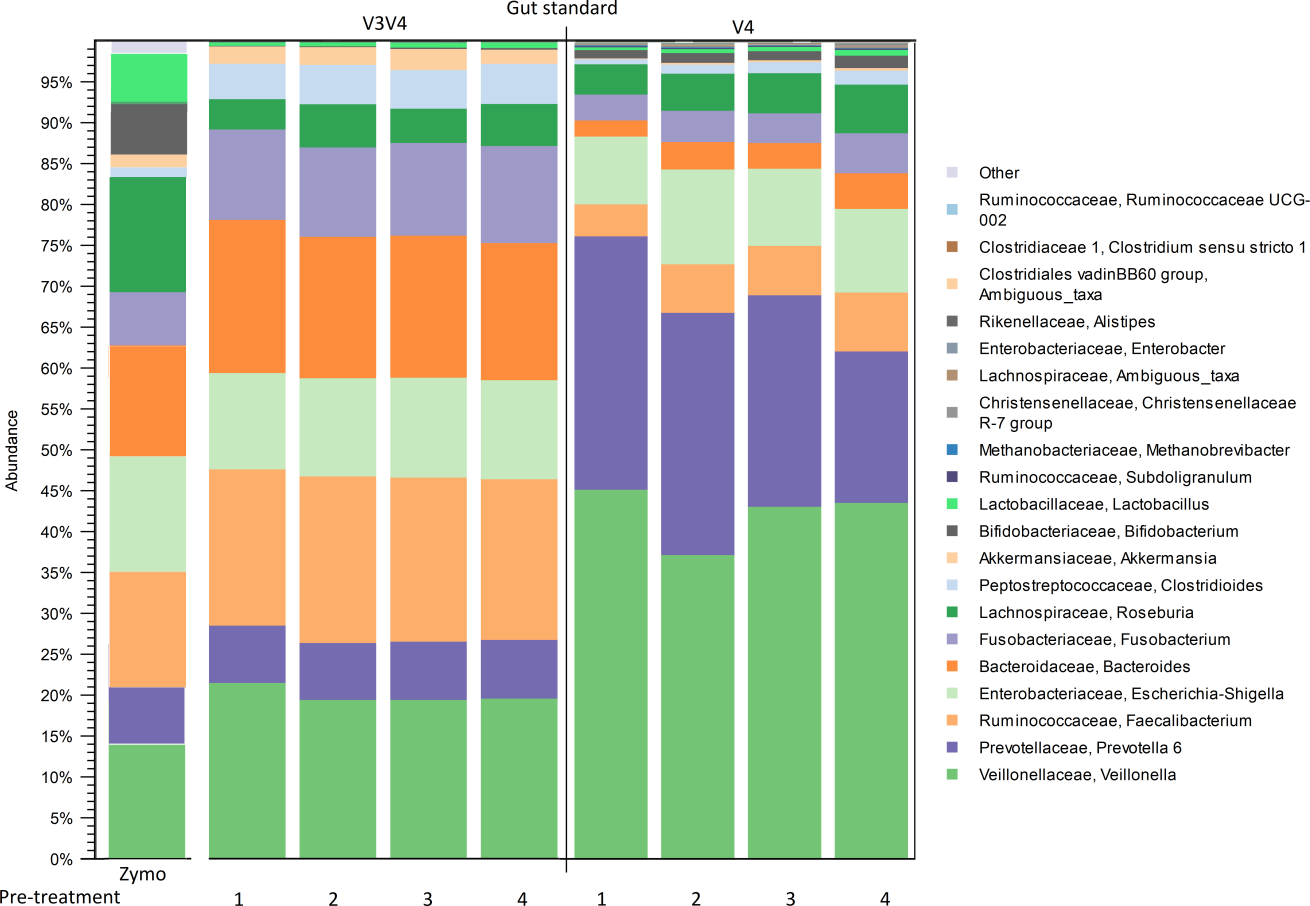
Relative abundance of Gut Standard across different pre-treatments with V3V4 and V4 sequencing. Numbers indicate pre-treatment groups (1–4). The manufacturer’s (Zymo) expected abundances are shown on the left of the figure. Legend shows the 20 most abundant genera.

In relation to the ZymoBIOMICS Gut Standard, the results were relatively similar across the pre-treatment groups within the same sequencing target. However, the V3V4 sequencing produced more similar results to the Gut Standard than V4, which notably differed from the manufacturer’s expected abundances (Fig. 2).

The V3V4 sequencing produced higher relative abundances of the genera *Fusobacterium, Clostridioides, Akkermansia, Bacteroides, Veillonella*, and *Prevotella* than the manufacturer stated. On the other hand, the genera *Bifidobacterium* and *Lactobacillus* were less abundant than expected. With the V3V4 sequencing, differences between the pre-treatments were minor and differences in relative abundances fluctuated between 1% and 2%.

V4 sequencing favored the genera *Veillonella and Prevotella* in comparison with the manufacturer’s reported values (Fig. 2). V4 sequencing detected five to six times higher relative abundance of *Prevotella* and a twofold higher abundance of *Veillonella* than the manufacturer’s reported abundances. The genera *Faecalibacterium, Roseburia, Bacteroides, Fusobacterium, Clostridioides, Akkermansia, Bifidobacterium*, and *Lactobacillus* were lower in abundance in comparison with the Gut Standard. The V4 sequencing results had more variation between the pre-treatment groups (1%–10%) than the V3V4 sequencing. The genera *Veillonella* and *Prevotella* decreased in abundance from the non-bead-beating groups (1 and 2) to the bead-beating groups (3 and 4). Subsequently, the genera *Bacteroides, Faecalibacterium, Roseburia, Lactobacillus*, and *Bifidobacterium* increased in relative abundance with bead beating (groups 3 and 4). The relative abundance of *Escherichia* stayed consistent across the pre-treatment groups and sequencing targets.

Both sequencing target areas favored the genera *Veillonella* and *Prevotella* in relation to the manufacturer’s result, whereas the genera *Bifidobacterium* and *Lactobacillus* were lower in abundance. *Enterococcus* was detected in all samples in the V3V4 sequencing. With the V4 sequencing, *Enterococcus* was seen only in pre-treatment groups 3 and 4. Differences between replicates in relative abundances fluctuated between 0% and 1% with V3V4, and 1% and 4% with V4. In addition to the expected genera, V4 detected 121 and V3V4 detected 31 additional genera, with an abundance of under 1%.

### Fecal samples: relative and differential abundances

The adult fecal samples were dominated by the phyla *Firmicutes*, followed by *Bacteroidetes* and *Actinobacteria*. The profiles were relatively similar for both sequencing targets. Bead beating (groups 3 and 4) added signatures from hard-to-lyse gram-positive genera such as *Blautia, Bifidobacterium*, and *Ruminococcus torques* group (Fig. S2). Samples with DNA/RNA shield fluid had a higher abundance of *Faecalibacterium*. Samples with OMNIgeneGUT showed a higher abundance of Bacteroides. The effect of bead beating was not as visible when DNA/RNA shield fluid was utilized.

For senior fecal samples, the most abundant phyla were *Firmicutes*, *Bacteroidetes*, *and Proteobacteria* (Fig. S3). The profiles were relatively similar within the same sequencing target; however, V3V4 and V4 differed from each other. Genus *Klebsiella* from family *Enterobacteriaceae* was missing in V4 sequenced samples, and genus *Enterobacter* was missing in V3V4 sequenced samples.

The most abundant phyla in the infant samples were *Bacteroidetes*, *Firmicutes*, and *Proteobacteria*. V3V4 sequencing favored genera *Bacteroides* and *Klebsiella*, whereas *Veillonella* and *Enterobacter* were more abundant with the V4 sequencing (Fig. S4). There were minor increases in the abundances of *Enterobacter and Pantoea* genera in bead-beating groups 3 and 4 in the V4 sequencing. The profiles were relatively similar within the same sequencing target. The effect of the target region had a high impact on the infant sample. The profiles were dominated with the same bacteria but the ratios differed greatly.

**Fig 3:**
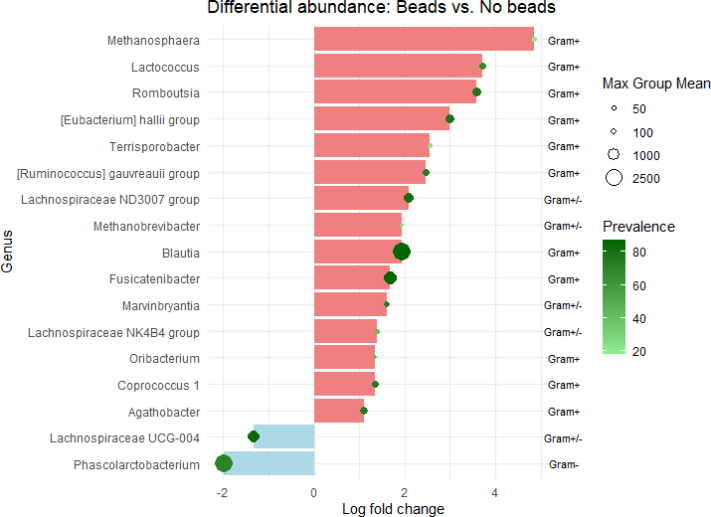
The effect of bead beating. Differential abundance analysis with fecal samples, comparison of bead beating versus no bead beating. X-axis shows log2 fold change. Maximum group means (abundances) are illustrated with different dot sizes, and prevalence of genus by color scale (green). The figure summarizes the consensus of CLC and Deseq2 results. These differences were significant (FDR-*P* ≤ 0.05).

Taken together, differential abundance analysis (DAA) of fecal samples (Fig. 3) shows that bead beating increased the abundances of several gram-positive bacteria. These differences were significant (FDR-*P* ≤ 0.05); however, DAA has limitations, particularly in the context of high inter-individual variation in the microbiome, which may explain the high variability in abundances and prevalence. Figure 3 shows the genera that were both found by the CLC and Deseq2.

### Alpha diversities

**Fig 4:**
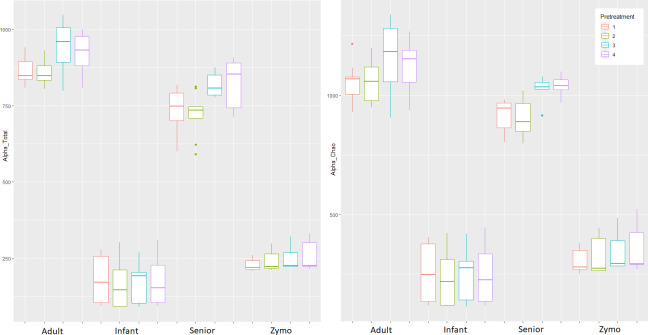
Alpha diversity indexes in boxplots by observed OTUs (left) and Chao1 (right) across sample types and pre-treatment groups. Boxplots show minimum, first quartile (Q1), median, third quartile (Q3), and maximum and outliers.

The alpha diversity indexes were calculated based on observed OTUs and Chao1 (Fig. 4). Both indexes showed similar results. In adult and senior samples, the bead-beating groups 3 and 4 had higher alpha diversity levels than the groups without bead beating (adult *P* = 0.0003, senior *P* = 0.0017). The highest alpha diversity in the adult samples was in group 3, and in the senior samples in group 4. Both bead-beating groups had higher variation compared to the non-bead-beating groups (1 and 2) in adult samples. Senior samples had less variation in the bead-beating groups in Chao1 metric.

With the infant samples, on average, the bead-beating group 3 produced the highest diversity. The variation between the replicates was relatively high. Overall, the effect of pre-treatment was smaller in the infant samples compared to the adult and senior samples. Similarly, with the Gut Standard, the effect of pre-treatment was minor. Group 3 and 4 produced a slightly higher alpha diversity. In the Gut Standard and infant samples, the difference between bead-beating and non-bead-beating groups was not statistically significant.

### Beta diversities

**Fig 5:**
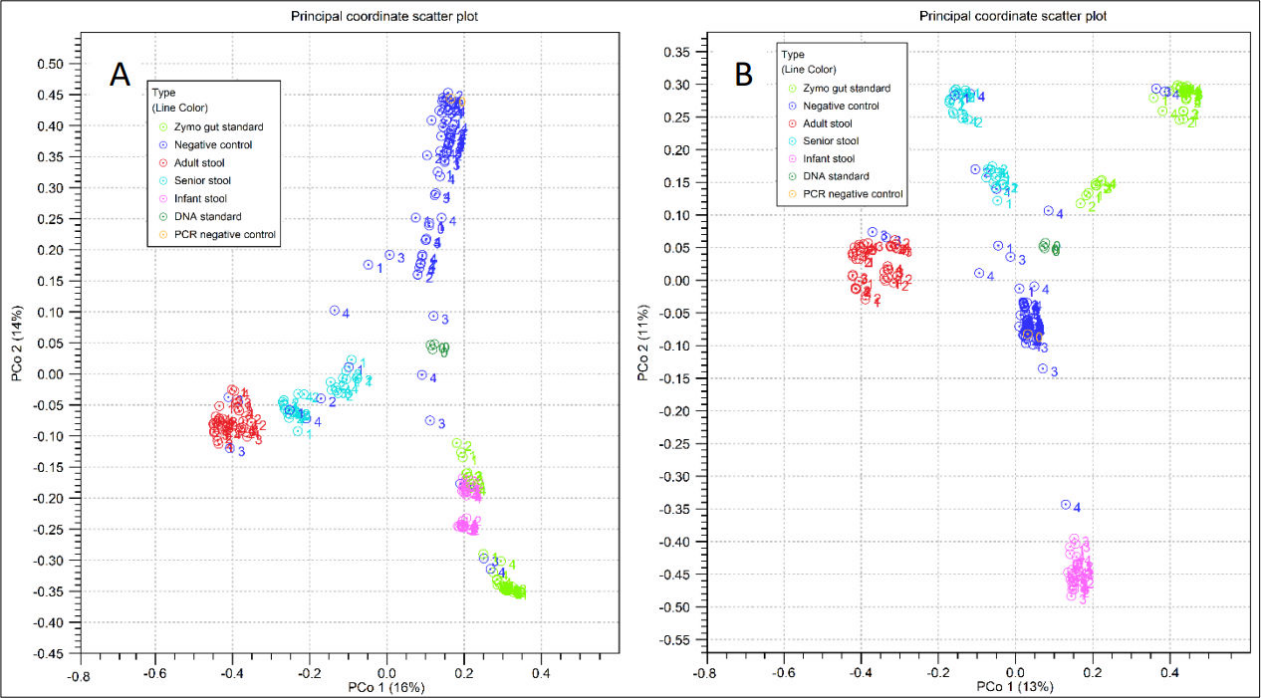
Beta diversity by Bray-Curtis (**A**) and Jaccard (**B**) among all sample types. Sample types are listed in legend. Numbers indicate pre-treatment groups 1–4.

Figure 5 summarizes the beta diversities between all the sequenced samples using Bray-Curtis (**A**) and Jaccard (**B**) distance metrics. All the sequenced samples (total = 192) clustered in their own groups in exception of seven negative controls, which were located in clusters of fecal samples or the Gut Standard. There were no shifts from one fecal sample group to another. Bead beating did not increase the read count (*P* = 0.36). Moreover, grouping by the 16S target region (V3V4 and V4) can be seen within the subject clusters (Fig. S5).

**Fig 6:**
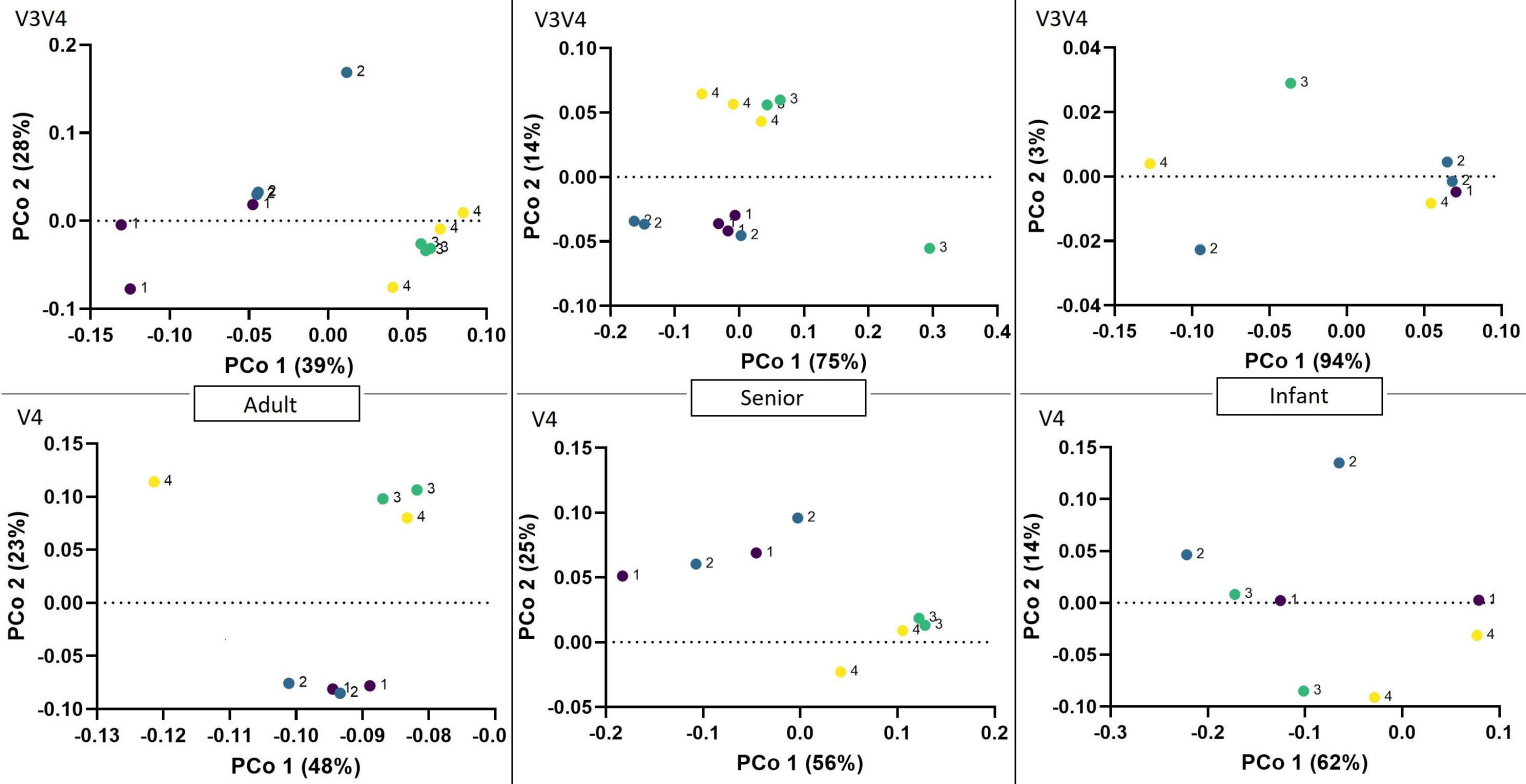
Beta diversities of fecal samples in V3V4 and V4 across different pre-treatment groups with Bray-Curtis measure. Pre-treatments groups are indicated with differently colored dots (1, purple; 2, blue; 3, green; 4, yellow) and numbers adjacent to the dots.

Bead-beating samples without proteinase K incubation (3) and with proteinase K incubation (4) are loosely grouped in the adult and senior fecal samples (Fig. 6). Similarly, the chemical lysis samples with proteinase K incubation (1) and without proteinase K (2) are loosely grouped. Both sequencing methods exhibit similar trends in the pre-treatment grouping. However, the V4 sequenced senior samples seemed to have more variation within groups 1 and 2. Infant fecal samples did not exhibit specific grouping across the pre-treatment groups.

### Negative controls

**Fig 7:**
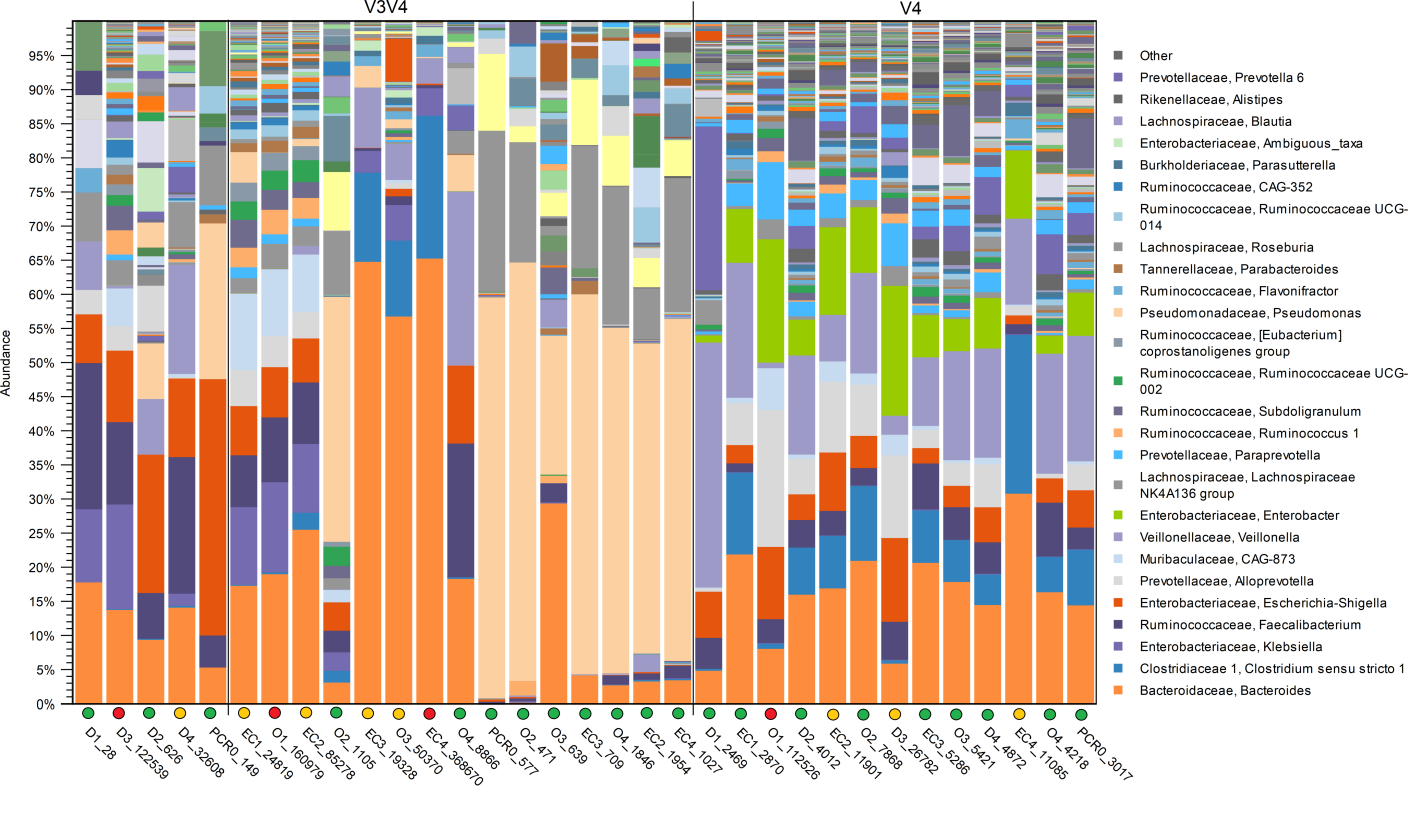
Relative abundances of V3V4 and V4 sequenced negative controls, DNA/RNA shield fluid (D), OMNIgeneGUT (O), Lysis Buffer 1 as extraction controls (EC), and PCR blanks (PCR0). Extraction groups are indicated with numbers 1–4. Numbers after the underscore are reads in OTUs. Colored circles indicate the quantity of reads in OTUs (green < 10,000, yellow 10,000–100,000, red > 100,000).

Negative controls were included in analysis to detect possible cross-contamination (Fig. 7). The negative controls with V3V4 sequencing were dominated by genera present in the fecal samples, such as *Bacteroides*, *Faecalibacterium*, and *Klebsiella*. Approximately half of the V3V4 sequenced negative controls were dominated by the genus *Pseudomonas*, and those profiles were similar to the PCR-0-control, which contained only PCR-grade water. The PCR-0 controls in V3V4 were dominated by the genera of *Pseudomonas*, *Serratia*, *and Delftia*, which were not commonly detected in the fecal samples. Accordingly, the negative controls within V4 sequencing were dominated by genera present in the fecal samples, such as *Bacteroides*, *Veillonella*, and *Alloprevotella*. The V3V4 sequenced negative controls had more variation in their profiles, whereas the V4 results were more uniform.

The V3V4 sequenced negative controls had, on average, 53,203 reads of trimmed pairs. The V4 sequenced controls had, on average, 27,387 reads of trimmed pairs. The individual read counts of the negative and extraction controls are shown in Table 2. The V3V4 sequenced controls had a higher read count and more variation than those sequenced by V4.

**TABLE 2:** Read counts of negative controls with V4 and V3V4^*a*^

Negative controls, V4, read counts (paired, trimmed pairs)					
ID	Reads	ID	Reads	ID	Reads
DNA shield 1_1	✓ 2,863	Omni 1_1	*✓* 15,641	EC 1_1	*✓* 5,415
DNA shield 1_2	✓ 3,271	Omni 1_2	!! 123,738	EC 1_2	*✓* 5,864
DNA shield 2_1	! 26,052	Omni 2_1	*✓* 15,595	EC 2_1	! 34,811
DNA shield 2_2	! 34,934	Omni 2_2	! 25,796	EC 2_2	! 33,879
DNA shield 3_1	! 40,892	Omni 3_1	! 39,777	EC 3_1	! 57,900
DNA shield 3_2	! 54,439	Omni 3_2	! 36,932	EC 3_2	*✓* 15,211
DNA shield 4_1	*✓* 21,053	Omni 4_1	*✓* 19,867	EC 4_1	*✓* 2,427
DNA shield 4_2	! 31,600	Omni 4_2	*✓* 6,395	EC 4_2	*✓* 16,672
PCR-0_V4	*✓* 13,657				

Negative controls, V3V4, read counts (paired, trimmed pairs)					
ID	Reads	ID	Reads	ID	Reads
DNA shield 1_1	! 85,290	Omni 1_1	*✓* 5,857	EC 1_1	*✓* 4,367
DNA shield 1_2	*✓* 2,188	Omni 1_2	!! 261,396	EC 1_2	! 41,043
DNA shield 2_1	*✓* 150	Omni 2_1	*✓* 4,526	EC 2_1	*✓* 2,956
DNA shield 2_2	*✓* 108	Omni 2_2	*✓* 2,576	EC 2_2	!! 148,071
DNA shield 3_1	!! 200,210	Omni 3_1	!! 238,062	EC 3_1	!! 239,797
DNA shield 3_2	*✓* 856	Omni 3_2	! 77,678	EC 3_2	! 29,192
DNA shield 4_1	*✓* 565	Omni 4_1	*✓* 4,286	EC 4_1	*✓* 2,511
DNA shield 4_2	! 64,789	Omni 4_2	*✓* 14,545	EC 4_2	!! 553,171
PCR-0_V3V4_1	*✓* 1,427				
PCR-0_V3V4_2	✓ 888				

^*a*^ DNA shield, DNA/RNA shield fluid; Omni, OMNIgeneGUT; EC, extraction control/lysis buffer; first number, pre-treatment group; second number, number of replicate, e.g., EC1_2 is an extraction control with pre-treatment 1 and second replicate. The symbols indicate the number of reads: ✓ < 25,000; 25,000 < **!** < 100,000; **!!** > 100,000 reads.

**Fig 8:**
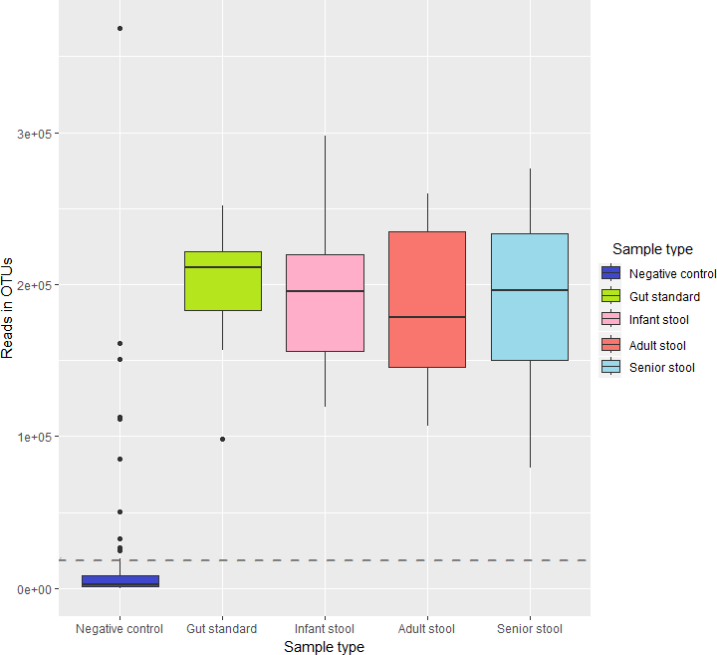
Number of reads in OTUs in different sample types. The horizontal lines inside boxplots indicate the average in a sample type. The horizontal dotted lines reflect the QC fraction.

The number of reads in OTUs across the different sample types can be seen in Figure 8. Although there were a relatively high number of reads in the negative controls, the level was still notably lower compared to the actual samples. If a threshold is needed in quality control, those should be based on relative fraction in read counts (reads in OTUs or total read count). Based on the reads in OTUs, the QC fraction (arithmetic mean of read counts in negative per positive) was 12%, but it should be noted that negative samples had a skewed read count distribution.

## DISCUSSION

In this study, we applied several methodological approaches to explore the optimization of a high-throughput stool pre-treatment and DNA isolation method suitable for downstream microbiome sequencing analyses. We established a pre-treatment protocol, which includes bead-beating step followed by automated DNA extraction with Chemagic DNA Stool kit, both in 96-format.

Bead beating yielded a higher microbial diversity and the increase of harder-to-lyse gram-positive bacteria in the fecal sample profiles, as previously reported (22, 48). The senior and adult fecal samples showed the highest alpha diversities with bead beating. Infant fecal samples showed variable microbial diversities across the pre-treatment groups, indicating that infant samples do not benefit from bead beating as much as senior and adult samples, because infant gut microbiota is not as rich. It seems that the higher the diversity, the higher the impact of the bead beating. The isolation protocol was also evaluated by using the Gut Microbiome Standard. The observed compositions of the Gut Standard samples differed considerably from the expected theoretical compositions in all the methods. Furthermore, the compositions of Gut Standard showed minor variability between the pre-treatment methods, as well as considerable variation between the sequenced 16S variable regions.

Both preservatives, OMNIgeneGUT and DNA/RNA shield, were usable for DNA extraction and sequencing. The replicates from DNA/RNA shield fluid were more comparable and this might be caused by the 1:10 ratio of sample and preservative, whereas OMNIgeneGUT had a ratio of 1:4. The microbiome profiles showed minor differences between preservatives. The DNA shield had higher abundances of *Faecalibacterium* while OMNIgeneGUT showed higher abundances of *Bacteroides*. These results are in line with Chen et al. (25). OMNIgeneGUT tubes were more practical in terms of sample collection; the correct sample volume was easy to collect, and the mixing was convenient with metal bead in the tube. OMNIgeneGUT can be convenient in field studies with at-home-collected samples and it has been used successfully in several studies (49–51).

The 16S variable target region had a high impact on the microbial compositional profiles as has already been reported (45, 52–54). However, differences in family and genus abundances occurred in similar proportions in the different pre-treatment groups. The impact of the variable region was highest with the infant sample and lowest with the adult sample. Different sequencing targets seemed to favor different bacterial genera. Indeed, the sequencing region seemed to have more impact on the compositional profiles than the pre-treatment. It seemed that the lower the diversity, the higher the impact of the V-target region. It was expected that sequencing with V3V4 would produce more results similar to the Gut Standard because the same target area was used by the manufacturer (55). To reach more rigorous quality control, the use of spike-in controls could be beneficial when controlling the resolution of the DNA extracts and the quality of the libraries. Detection thresholds are challenging to set, and they should be calculated based on individual runs and sample type and can be highly variable between laboratories. Because the NGS methods are prone to contamination, the possibility of contamination and a need of reruns should be considered, if the read count in negative samples rises above the relative QC fraction (~10%).

The selected protocol included OMNIgeneGUT tubes for sample collection, pre-treatment with bead beating and proteinase K incubation (group 4), a Chemagic stool kit, and an MSM1 extraction robot. The turnaround time for the selected pre-treatment was long, because pre-treatment 4 included bead beating and proteinase K incubation. Moreover, pre-treatments 3 and 4 were more expensive, because bead plates were not included in the extraction kit. Proteinase K was estimated to be necessary to avoid DNases and inhibitors when DNA is stored for a longer period. Manual extraction kits were not tested or compared with Chemagic, because the aim was to an automated method with reduced hands-on time. Based on its DNA yield, efficient recovery of DNA from gram-positives, and overall small bias, as well as its reasonable turnaround time and cost, we selected pre-treatment 4 as the basis for our standard operating procedure (SOP) for DNA extraction. To reduce hands-on time, adjustable multi-channel pipettes and a plate format thermomixer are utilized in further extractions for upcoming studies with large sample collections. To assist the pipetting of fecal material, OMNIgene Liquefaction (DNA Genotek, Canada) can be utilized to make samples less viscous. Chemagic 360 instrument (PerkinElmer, Germany), the next version of MSM1, can also be used for DNA extractions with identical principal and the same stool kit. Bioanalyzer, Tapestation, or a similar device should be utilized in quality control, especially when approaching shotgun sequencing.

The relatively high read count of the negative controls can be explained by the 96-well plate format; the samples are close to each other, and aerosols cannot be fully avoided. Cross-contamination was observed from the negative control read counts, the relative abundances of the controls, and beta diversity resembling those of the fecal samples. V3V4 can be more problematic due to two PCR steps and more library preparation steps where contamination might be introduced. Amplicon PCR product purification with V3V4 is particularly susceptible to contamination, because sample-specific indexes have not yet been added. Correspondingly, V4 has only one PCR step, and the sample-specific indexes have been added to the primers.

Due to the variable consistency of the fecal samples, it can be challenging to obtain an equal amount of sample for all replicates. Fecal matter is proven to have a heterogenous consistency of a semi-solid mixture of endogenous and exogenous material (56), and these together might explain the variation within replicates. This variation relates to the resolution of the protocol (fluctuation in relative abundance: V3V4 0%–1% and V4 1%–4%), and it also indicates error rate, which should be considered when analyzing low abundances. Technical replicates are needed to estimate not only the degree of variation but also the existence of contamination. This was a pilot study for a DNA extraction in a large human cohort. Due to the low sample size, statistical analyses were limited. In further studies, a larger sample size is recommended for the statistical power that is needed to observe the effect of different methodological choices.

### Conclusion

The applied 96-format extraction system, including the sample pre-treatment steps, proved to be a functional workflow in stool DNA extraction in clinical microbiome research. The sequencing target region effect was larger than the effect of the pre-treatment method. It is notable that the choice of the sample preservative, bead beating, and 16S target region have a varying effect on microbiome profiles. These results indicate the need for standardized methods for microbial profiling. Therefore, we propose the following points to achieve best practice: consider using a well-established preservative to maintain microbial integrity during storage, utilize a 96-format extraction system coupled with bead beating for efficient and high-throughput stool DNA extraction, and control the level of contamination and bacterial coverage of chosen methods.

## Data Availability

Sequence data (FastQ-files) and metadata have been deposited into the SRA database under the BioProject PRJNA955433. Supplementary figures and tables are available in a Word document and Excel files accompanying the manuscript. STORMS checklist is available at: https://figshare.com/s/a14e81de02317b2b7580.

## SUPPLEMENTAL MATERIAL

Supplemental figures and table spectrum.02932-23-s0001.docx Fig. S1–S5; Table S1.

Table S2 spectrum.02932-23-s0002.xlsx 6,000 genera (used for negative controls).

Table S3 spectrum.02932-23-s0003.xlsx 450 genera (used for fecal samples and positive controls).

Table S4 spectrum.02932-23-s0004.xlsx OTU table of relative abundances.

Table S5 spectrum.02932-23-s0005.xlsx Table of sample metadata and read counts.

ASM does not own the copyrights to Supplemental Material that may be linked to, or accessed through, an article. The authors have granted ASM a non-exclusive, world-wide license to publish the Supplemental Material files. Please contact the corresponding author directly for reuse.
