# Supplementary material for: High-throughput DNA extraction strategy for fecal microbiome studies
Source: Microbiol Spectr. 2024 May 15;12(6):e02932-23. doi: 10.1128/spectrum.02932-23 (PMC11237708; doi:10.1128/spectrum.02932-23)
Supplement: Supplemental figures and table — Fig. S1–S5; Table S1. [file spectrum.02932-23-s0001.docx]

Supplementary tables and figure

Table S1. The average DNA concentrations

Figure S1. DNA isolates in gel electrophoresis

Figure S2. Relative abundance of V3V4 and V4 sequenced adult fecal samples across different pre-treatment groups and preservatives

Figure S3. Relative abundance of V3V4 and V4 sequenced senior fecal samples across different pre-treatment groups and preservatives.

Figure S4. Relative abundance of V3V4 and V4 sequenced infant fecal samples across different pre-treatment groups and preservatives.

Figure S5. Beta diversities in adult, senior and infant samples

Table S1. The average DNA concentrations and standard deviations (ng/µl) across different pre-treatment groups (1-4) and preservatives (OMNIgeneGUT and DNA/RNA shield).

| Samples | 1 | 2 | 3 | 4 | Average all | Stdev all |
| --- | --- | --- | --- | --- | --- | --- |
| Adult OMNIgeneGUT (AO) | 28.8 | 31.1 | 26.2 | 23.2 | 27.3 | 3.0 |
| Adult DNA/RNA Shield (AD) | 29.9 | 33.2 | 24.7 | 27.3 | 28.8 | 3.1 |
| Senior OMNIgeneGUT (SO) | 46.6 | 53.7 | 47.4 | 55.3 | 50.8 | 3.8 |
| Senior DNA/RNA Shield (SD) | 36.1 | 15.0 | NA | NA | 25.6 | 10.5 |
| Infant OMNIgeneGUT (IO) | 32.1 | 33.5 | 11.7 | 8.1 | 21.3 | 11.5 |
| Infant DNA/RNA Shield (ID) | 25.1 | 18.6 | 8.1 | 7.1 | 14.7 | 7.5 |
| Zymobiomics Gut Standard | 6.4 | 6.0 | 4.9 | 4.0 | 5.4 | 1.0 |


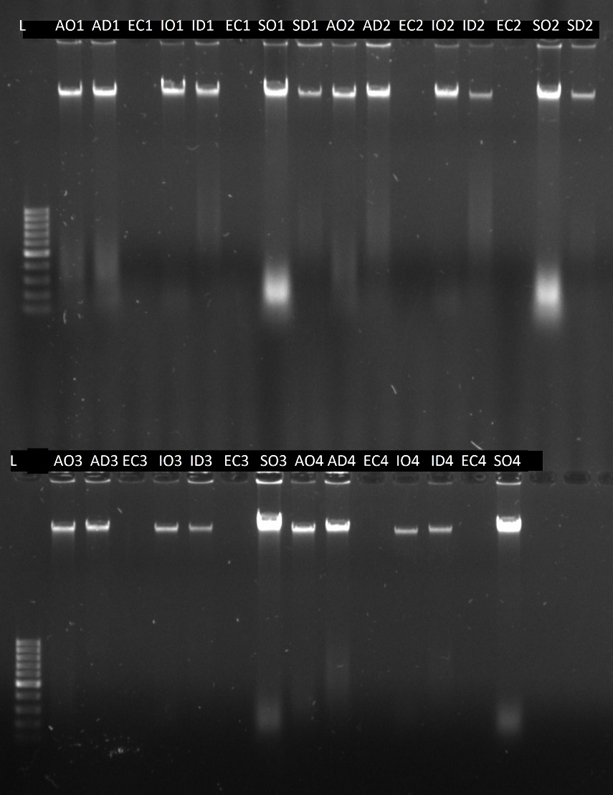


*Figure S1. DNA isolates in gel electrophoresis. Letters: A=adult, S=senior, I=infant, O=OMNIgeneGUT, D= DNA/RNA shield, EC= extraction control/negative, L=ladder, numbers 1-4 = pre-treatment groups.*


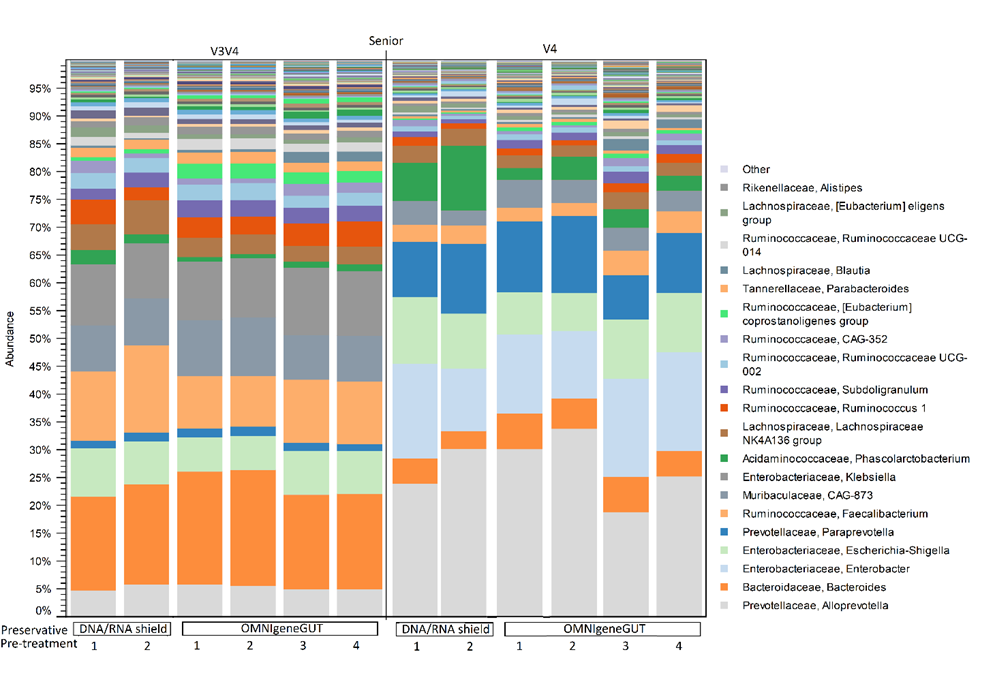

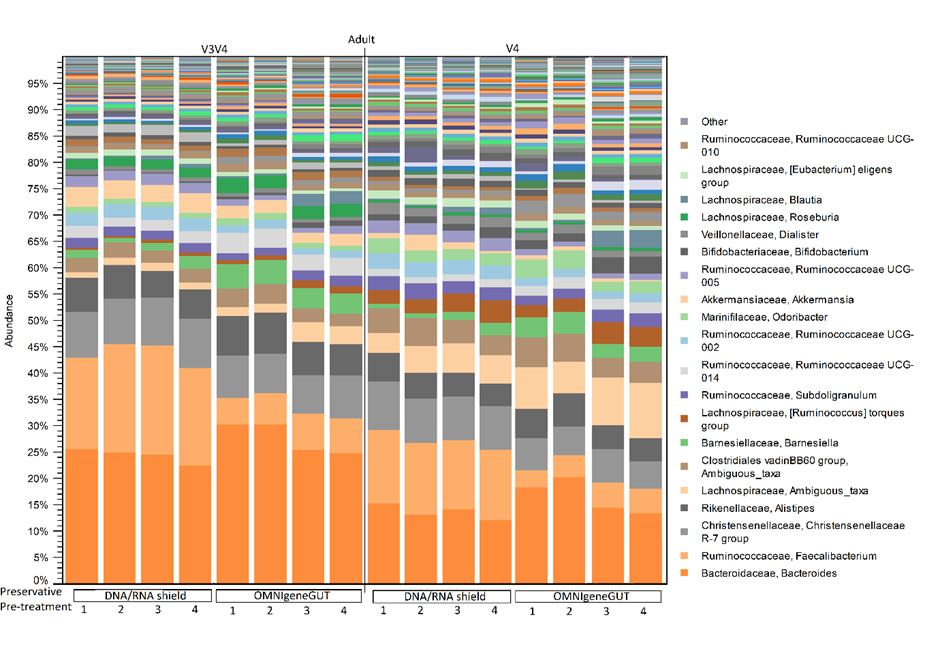
 Figure S2. Relative abundance of V3V4 and V4 sequenced adult fecal samples across different pre-treatment groups and preservatives. Legend shows 20 most abundant genera.

*Figure S3.* *Relative abundance of V3V4 and V4 sequenced senior fecal samples across different pre-treatment groups and preservatives. Legend shows 20 most abundant genera.*


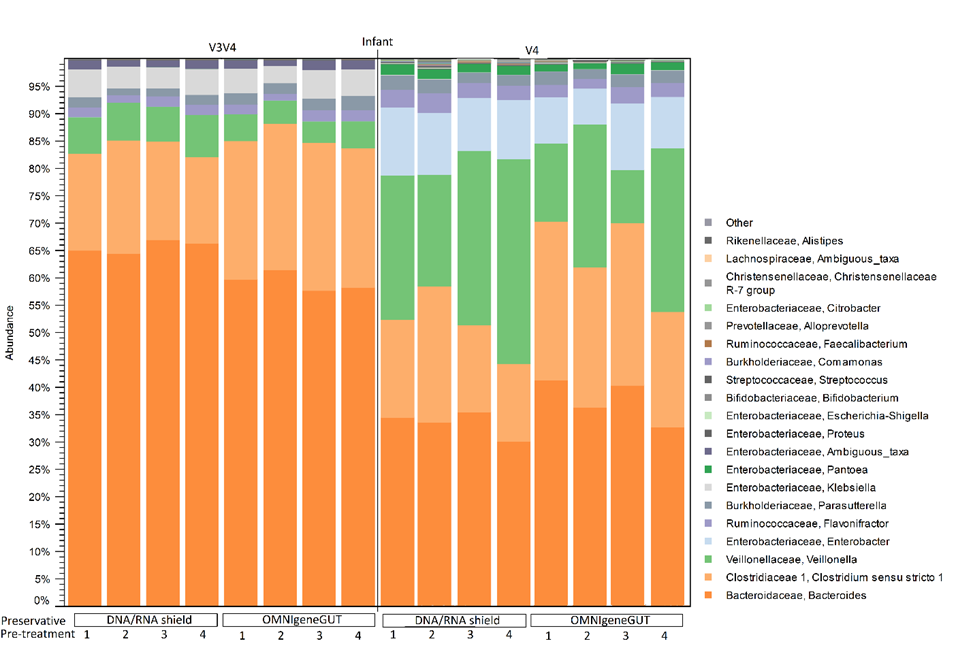


*Figure S4.* *Relative abundance of V3V4 and V4 sequenced infant fecal samples across different pre-treatment groups and preservatives. Legend shows 20 most abundant genera.*


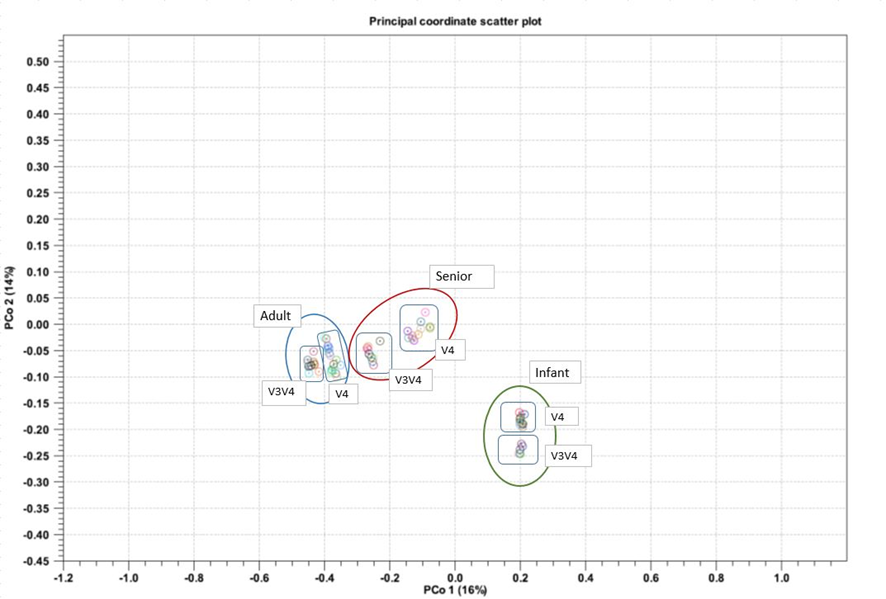


*Figure S5. Beta diversities in adult, senior and infant samples with different 16S target regions (V3V4 and V4) by Bray-Curtis.*
